# β2-Microglobulin Regulates Extracellular Matrix Dynamics During Peripheral Nerve Injury

**DOI:** 10.3390/neurosci6030059

**Published:** 2025-06-29

**Authors:** Eiki Shirasawa, Kentaro Uchida, Kenji Onuma, Gen Inoue, Koji Eshima, Masashi Satoh, Masayuki Miyagi, Yoji Toyomura, Akira Norisugi, Masashi Takaso

**Affiliations:** 1 Department of Orthopaedic Surgery, Kitasato University School of Medicine, Sagamihara 252-0374, Kanagawa, Japan; eeiikkii922@yahoo.co.jp (E.S.); onuma@med.kitasato-u.ac.jp (K.O.); ginoue@kitasato-u.ac.jp (G.I.); masayuki008@gmail.com (M.M.); toyomura.yoji@st.kitasato-u.ac.jp (Y.T.); norisugi.akira@st.kitasato-u.ac.jp (A.N.); mtakaso@kitasato-u.ac.jp (M.T.); 2 Medical Sciences Research Institute, Shonan University, Chigasaki 253-0083, Kanagawa, Japan; 3 Division of Immunology, Department of Biosciences, Kitasato University School of Science, 1-15-1 Minami-ku, Kitasato, Sagamihara 252-0373, Kanagawa, Japan; eshima.koji@kitasato-u.ac.jp; 4 Department of Immunology, Kitasato University School of Medicine, 1-15-1 Minami-ku, Kitasato, Sagamihara 252-0374, Kanagawa, Japan; msato@med.kitasato-u.ac.jp

**Keywords:** β2-microglobulin, extracellular matrix, sciatic nerve, chronic constriction injury

## Abstract

Peripheral nerve injury initiates a complex cascade of events coordinating immune responses, extracellular matrix (ECM) remodeling, and neuronal repair. While β2-microglobulin (B2M) is well known for its role in MHC class I-mediated antigen presentation and CD8^+^ T-cell differentiation, its potential contributions to non-immune processes remain underexplored. In this study, we investigated the role of B2M in peripheral nerve regeneration using a chronic constriction injury (CCI) model in wild-type and B2M-deficient (B2M-KO) mice. Flow cytometry, RNA sequencing (RNA-seq), and quantitative PCR (qPCR) were performed to assess T-cell subset dynamics and gene expression following injury. Flow cytometric analysis showed that CD3^+^CD4^+^ and CD3^+^CD8^+^ T-cell populations increased by day 7 post-injury. While CD3^+^CD4^+^ T-cell expansion occurred in both groups, a significant increase in CD3^+^CD8^+^ T cells was observed only in wild-type mice. RNA-seq analysis at 3 days post-injury—prior to substantial T-cell accumulation—revealed marked downregulation of ECM-related genes in B2M-KO mice, including collagens, matrix-associated proteins, and other key ECM components. KEGG analysis identified suppression of ECM–receptor interaction, PI3K-Akt, and TGF-β signaling pathways. qPCR confirmed reduced expression of Thbs1 in B2M-KO mice. These findings suggest that B2M plays a critical, CD8^+^ T-cell-independent role in regulating ECM dynamics and regenerative signaling during early nerve repair, expanding the conceptual framework of B2M’s function beyond classical immune roles.

## 1. Introduction

Peripheral nerve injuries, including traumatic and compression neuropathies, affect millions worldwide and often lead to lasting sensory and motor deficits. Conventional treatments, such as surgical nerve repair and grafting, yield variable outcomes and frequently fail to fully restore function [1,2]. Understanding molecular regulators that orchestrate the regenerative microenvironment is therefore critical to developing adjunctive therapies.

Peripheral nerve injuries are generally classified into distinct categories based on the anatomical extent and pathophysiological features of the lesion: (1) nerve transection, characterized by complete discontinuity of the nerve trunk and necessitating surgical coaptation; (2) segmental nerve defects, involving partial loss of nerve tissue and often requiring reconstruction using autologous or synthetic grafts; and (3) chronic constriction injuries, marked by sustained mechanical compression that triggers prolonged inflammation, demyelination, and partial axonal degeneration [3]. To investigate these mechanisms in a reproducible manner, several preclinical models have been established. Among them, the chronic constriction injury (CCI) model of the sciatic nerve is widely employed due to its ability to induce partial, non-transecting injury that mimics clinical features of neuropathic conditions. Although the CCI model does not recapitulate complete axonal regeneration, it provides a robust platform for studying injury-induced molecular dynamics and maladaptive responses in the peripheral nervous system [4,5].

Peripheral nerve injury, such as that induced in the CCI model, evokes a multifaceted sequence of inflammatory responses, extracellular matrix (ECM) remodeling, and the activation of regenerative signaling cascades [6,7]. The ECM not only provides a physical scaffold for regenerating axons but also presents biochemical cues—through integrins, growth factor sequestration, and matricellular proteins—that regulate Schwann cell migration, macrophage activation, and axonal guidance [8,9,10]. Among these, collagens (e.g., types I, III, VI, XII, and XIV), fibrillin, and matricellular proteins like thrombospondins and tenascins orchestrate distinct yet overlapping facets of the repair process [8,9]. Additionally, the ECM modulates key signaling pathways, including transforming growth factor-beta (TGF-β) and phosphatidylinositol 3-kinase/Akt (PI3K-Akt), which are indispensable for tissue remodeling and functional recovery [11,12,13].

β2-microglobulin (B2M) is traditionally recognized as a structural subunit of the major histocompatibility complex (MHC) class I molecule, where it facilitates antigen presentation to CD8^+^ T cells and plays a central role in adaptive immunity [14,15]. Mouse models lacking B2M (B2M-KO) exhibit a profound deficiency in CD8^+^ T cells and have demonstrated delayed peripheral nerve regeneration, a phenomenon historically attributed to impaired cytotoxic T-cell responses [16]. However, emerging evidence suggests that B2M exerts functions beyond immune regulation. Recent studies have highlighted its role in stem cell maintenance and modulation of macrophage phenotypes, and collagen production [17,18]. Moreover, B2M expression is influenced by the TGF-β signaling pathway [19], implicating its potential involvement in broader tissue repair processes.

Given these insights, we hypothesize that B2M contributes to peripheral nerve repair through direct transcriptional regulation of ECM components, in addition to its known roles in immune cell biology. In this study, we investigated the molecular mechanisms associated with B2M-induced ECM gene expression during the early stages of peripheral nerve injury using a B2M knockout (B2M-KO) mouse model and transcriptomic analysis.

## 2. Materials and Methods

### 2.1. Sciatic Nerve Injury (SNI) Model

All animal procedures were approved by the Kitasato University School of Medicine Animal Care Committee (Protocol No. 2024-113) and conducted in accordance with the IASP Ethical Guidelines for Investigating Experimental Pain in Conscious Animals and the Guidelines for Proper Conduct of Animal Experiments by the Science Council of Japan. Male C57BL/6J (Charles River Laboratories, Atsugi, Japan) and B2M-KO mice (aged 7 weeks, weighing 24–26 g) were housed in a semi-barrier facility under controlled conditions (12 h light/dark cycle; 21–23 °C; 45–65% humidity) with ad libitum access to standard rodent chow (CRF-1, Oriental Yeast Co., Ltd., Tokyo, Japan). Animals were randomly assigned to experimental groups, and all investigators were blinded to the genotype during surgeries and analyses.

**Figure 1:**
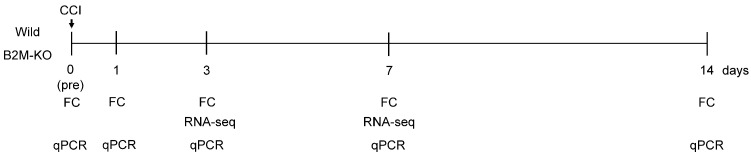
Experimental timeline illustrating sample collection and analysis following sciatic nerve injury in wild-type and B2M-KO mice.

SNI was induced using a CCI protocol adapted from previous studies [20,21]. Mice were anesthetized by intramuscular injection into the upper limbs of a mixture containing midazolam (Sand Co., Yamagata, Japan), Domitor™ (Nippon Zenyaku Kogyo Co., Ltd., Fukushima, Japan), and Vetorphale™ (Meiji Seika Kaisha, Ltd., Tokyo, Japan) in a ratio of 1:3:1 at a dose of 0.05 mL per 100 g body weight. The right sciatic nerve was exposed at the level of the mid-thigh just proximal to its trifurcation. Three loose ligatures with 9.0 nylon (Natsume Co., Ltd., Aichi, Japan) were placed around the SN at 1 mm intervals and tightened until a brief twitch was observed in the corresponding hind limb. Following ligation, the muscle and skin were carefully repositioned and closed in layers using 5-0 nylon sutures. SN samples were harvested the following day and at 1, 3, 7, and 14 days post-surgery. These time points were selected based on a previous report that evaluated T-cell infiltration dynamics in a CCI model of the mouse sciatic nerve [22]. The overall timeline of surgical procedures, tissue collection, and subsequent analyses is illustrated in Figure 1.

Chronic constriction injury (CCI) was induced in the right sciatic nerve of C57BL/6J (wild) and β2-microglobulin-deficient (B2M-KO) mice. Sciatic nerve (SN) tissues were harvested at five time points: pre-surgery (day 0) and at 1, 3, 7, and 14 days post-surgery. Flow cytometry (FC) and quantitative PCR (qPCR) analyses were performed at all five time points (days 0, 1, 3, 7, and 14). RNA sequencing (RNA-seq) was conducted at days 3 and 7.

### 2.2. Flow Cytometric Analysis

Sciatic nerves (*n* = 5 per group per time point) were minced and digested in 0.1% type I collagenase solution (Wako Pure Chemical Industries, Osaka, Japan) at 37 °C for 16 h with gentle agitation. Digests were filtered through 100-µm cell strainers (BD Falcon, Franklin Lakes, NJ, USA) and washed twice in ice-cold phosphate-buffered saline (PBS; Takara Bio, Otsu, Japan) by centrifugation at 300× *g* for 5 min. Cell suspensions were stained in staining buffer (PBS + 2% fetal bovine serum) with fluorochrome-conjugated monoclonal antibodies (all BioLegend, San Diego, CA, USA; clone and dilution indicated): PE/Cy5—anti-CD8a (clone 53-6.7, 1:100), PE—anti-CD4 (clone GK1.5, 1:100), PE/Cy7—anti-CD3ε (clone 145-2C11, 1:100), and APC/Cy7—anti-CD45 (clone 30-F11, 1:100). Samples were incubated for 30 min on ice in the dark, washed twice in PBS, and resuspended in 200 µL of PBS. After two PBS washes, samples were acquired on a BD FACSVerse cytometer. Data were acquired on a BD FACSVerse cytometer (BD Biosciences, San Diego, CA, USA), collecting at least 30,000 events per sample. Analysis was performed in FlowJo v10.1 (FlowJo LLC, Ashland, OR, USA) by gating on CD45^+^ cells and quantifying CD3^+^ subsets of CD4^+^ and CD8^+^ lymphocytes.

### 2.3. Transcriptome Analysis

Based on flow cytometry results indicating that T-cell recruitment becomes evident by day 7 post-injury, we selected day 3 (prior to T-cell infiltration) and day 7 (during T-cell accumulation) as time points for transcriptome analysis. Total RNA was extracted from sciatic nerve segments (~5 mm) as follows: nerves were submerged in TRIzol Reagent (Invitrogen, Carlsbad, CA, USA) on ice and homogenized using a Polytron^®^ homogenizer (KINEMATICA AG, Luzern, Switzerland) until uniform dispersion. Homogenates were centrifuged at 15,000 rpm for 10 min at 4 °C to pellet debris. The resulting supernatant was then applied to a Direct-zol RNA MicroPrep spin column (Zymo Research, Orange, CA, USA) for purification according to the manufacturer’s protocol, including on-column DNase I treatment. Purified RNA was eluted in RNase-free water. RNA concentration and purity were assessed using a spectrophotometer (Denovix, Wilmington, DE, USA), and RNA integrity was evaluated with an Agilent 2100 BioAnalyzer (Agilent Technologies, Santa Clara, CA, USA) using an RNA 6000 Nano-chip. RNA-seq libraries were prepared and sequenced on the DNBSEQ platform (BGI, Shenzhen, China), and subsequent data processing and analysis were conducted by BGI Japan. Differentially expressed genes (DEGs) were identified using a Q-value of ≤0.01 and a log_2_ fold change of ≥0.5. Pathway enrichment analysis was performed using the Kyoto Encyclopedia of Genes and Genomes (KEGG; http://www.genome.jp/kegg/, accessed 1 April 2024) through the BGI online analysis platform (Dr. Tom).

Among the ECM-related genes identified as differentially expressed in the RNA-seq analysis, we focused on thrombospondin-1 (Thbs1), which has been previously implicated in axon regeneration [10]. To validate the RNA-seq findings and investigate the temporal expression pattern of *Thbs1*, we performed quantitative real-time PCR (qPCR). For gene expression analysis, ten mice per group were anesthetized, and the ligated right sciatic nerve was harvested at baseline (day 0) and at 1, 3, 7, and 14 days post-surgery (each *n* = 10). Scar tissue was carefully removed from each nerve sample prior to RNA extraction. Total RNA was isolated using TRIzol reagent (Invitrogen, Carlsbad, CA, USA) according to the manufacturer’s instructions. First-strand cDNA was synthesized from total RNA using SuperScript III reverse transcriptase with DTT, First-Strand Buffer, random hexamer primers, and a dNTP mix, according to the manufacturer’s protocol (Invitrogen). qPCR was performed using gene-specific primers (see Table 1) on a CFX96 Real-Time PCR Detection System (Bio-Rad, Hercules, CA, USA). The amplification protocol consisted of an initial denaturation at 95 °C for 1 min, followed by 40 cycles of 95 °C for 5 s and 60 °C for 30 s. The expression level of *Thbs1* was normalized to glyceraldehyde-3-phosphate dehydrogenase (*Gapdh*) as an internal control.

### 2.4. Statistical Analysis

Data distribution was assessed using the Shapiro–Wilk test and was found to be non-normal. Consequently, non-parametric tests were applied: comparisons between two groups used the Mann–Whitney U test, and comparisons among three or more groups used the Kruskal–Wallis test with Dunn’s post hoc correction. All data are presented as box-and-whisker plots showing the median, interquartile range, and full range. Statistical analyses were performed using SPSS Statistics v28 (IBM, Armonk, NY, USA), and *p* < 0.05 was considered statistically significant.

## 3. Results

### 3.1. Differential Dynamics of CD4^+^ and CD8^+^ T-Cell Populations Between Wild-Type and B2M-KO Mice Following SNI

**Figure 2:**
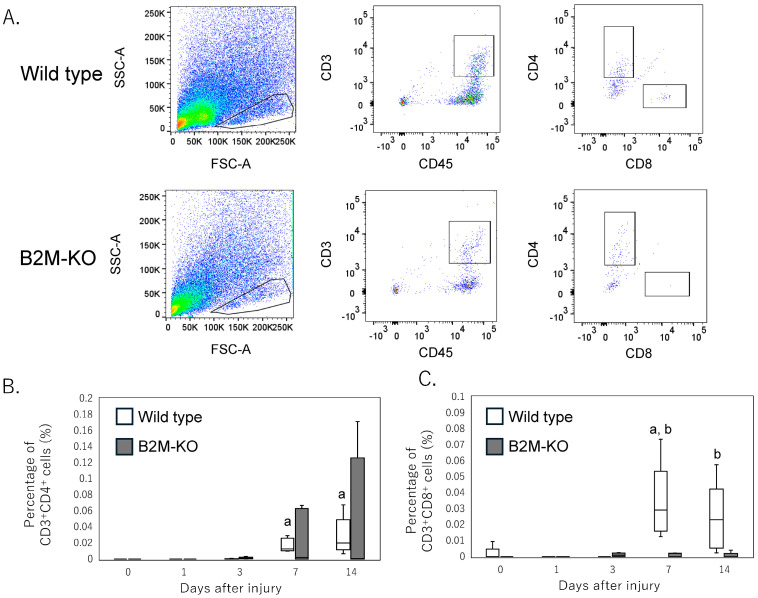
Flow cytometric analysis of T-cell subsets in the injured sciatic nerve following peripheral nerve injury. (**A**) Representative dot plots showing CD3^+^CD4^+^ and CD3^+^CD8^+^ T-cell populations in the injured sciatic nerve of wild-type and B2M-KO mice at day 7 post-injury. (**B**) Quantification of CD3^+^CD4^+^ T cells as a percentage of total live cells at days 0, 1, 3, 7, and 14 post-injury. (**C**) Quantification of CD3^+^CD8^+^ T cells at corresponding time points. Data are presented as box-and-whisker plots indicating the median, interquartile range, and minimum-to-maximum values. Statistical significance was determined using the Mann–Whitney U test for comparisons between wild-type and B2M-KO mice at each time point and the Kruskal–Wallis test with Bonferroni correction for comparisons across time points within each genotype. *p* < 0.05. (a) Significant difference compared to day 0 within each genotype. (b) Significant difference between wild-type and B2M-KO mice at the same time point.

Flow cytometric analysis revealed that the proportion of CD3^+^CD4^+^ T cells in wild-type mice remained unchanged at early time points (day 1 and day 3) but significantly increased at days 7 and 14 post-injury compared with day 0 (day 7, *p* = 0.036; day 14, *p* = 0.012) (Figure 2A,B). Similarly, B2M-KO mice exhibited an upward trend in CD3^+^CD4^+^ T cells at days 7 and 14; however, this increase did not reach statistical significance relative to day 0 (day 7, *p* = 0.055; day 14, *p* = 0.055) (Figure 2A,B). No significant differences in CD3^+^CD4^+^ T-cell populations were observed between wild-type and B2M-KO mice at any of the time points assessed.

In contrast, the CD3^+^CD8^+^ T-cell subset significantly increased at day 7 post-injury in wild-type mice compared with day 0 (*p* = 0.045), whereas B2M-KO mice exhibited no significant alterations in this subset throughout the observation period (Figure 2A,C). Furthermore, the proportion of CD3^+^CD8^+^ T cells was significantly higher in wild-type mithan in B2M-KO mice at day 7 (*p* = 0.008) and day 14 (*p* = 0.016) (Figure 2A,C).

### 3.2. Altered Extracellular Matrix Dynamics and Remodeling Pathways in B2M-KO Mice Following SNI

**Figure 3:**
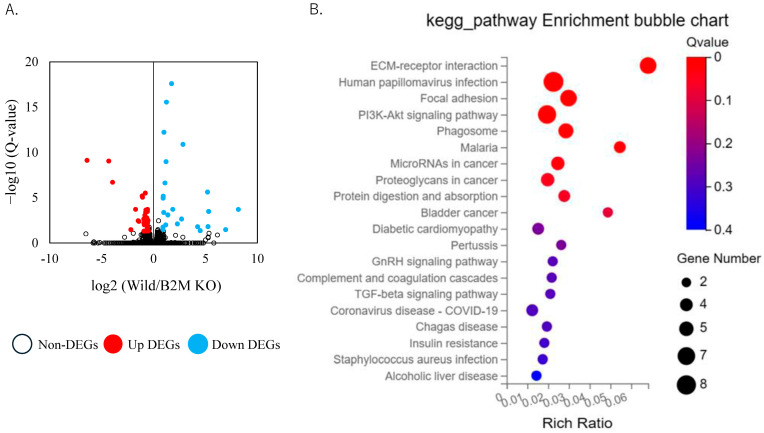
Transcriptomic changes in the sciatic nerve at day 3 post-injury. (**A**) Volcano plot depicting differentially expressed genes (DEGs) in the sciatic nerve at day 3 post-injury. Light blue dots represent genes significantly downregulated in B2M-KO mice compared to C57BL/6J (wild) mice; red dots indicate genes significantly upregulated in B2M-KO mice; and white dots denote non-differentially expressed genes. (**B**) KEGG pathway enrichment analysis of genes significantly downregulated in B2M-KO mice at day 3 post-injury.

Based on flow cytometric analysis results, we performed transcriptome profiling of sciatic nerves at day 3 (prior to CD8^+^ T-cell recruitment) and day 7 (post CD8^+^ T-cell recruitment). RNA sequencing at day 3 post-injury revealed significant differences between B2M-KO mice and wild-type (WT) controls, with 26 upregulated and 43 downregulated differentially expressed genes (DEGs) identified (Figure 3A, Supplementary Table S1). Notably, we observed pronounced downregulation of genes associated with ECM composition and remodeling in B2M-KO mice (Table 2). Specifically, collagen isoforms, including *Col12a1* and *Col14a1*, were markedly reduced. Additionally, matrix-associated proteins such as fibrillin 1 (*Fbn1*), latent transforming growth factor-beta binding protein 4 (Ltbp4), and microfibril-associated protein 1a (Mfap1a) showed significantly decreased expression levels.

Moreover, several structural ECM components, particularly members of the thrombospondin family (Thbs1 and Thbs3) and tenascins (Tnc and Tnxb), exhibited significantly lower expression in the B2M-KO group. The matrix remodeling enzyme matrix metalloproteinase-2 (Mmp2) was also notably downregulated. KEGG pathway enrichment analysis at this early time point further demonstrated significant enrichment and downregulation of genes involved in ECM–receptor interaction, PI3K-Akt signaling, and TGF-beta signaling pathways in B2M-KO mice compared with WT controls (Figure 3B).

DEGs were identified using a threshold of Q-value < 0.01 and log_2_ fold change ≥ 0.5. KEGG analyses were performed using the BGI online analysis platform (Dr. Tom).

**Figure 4:**
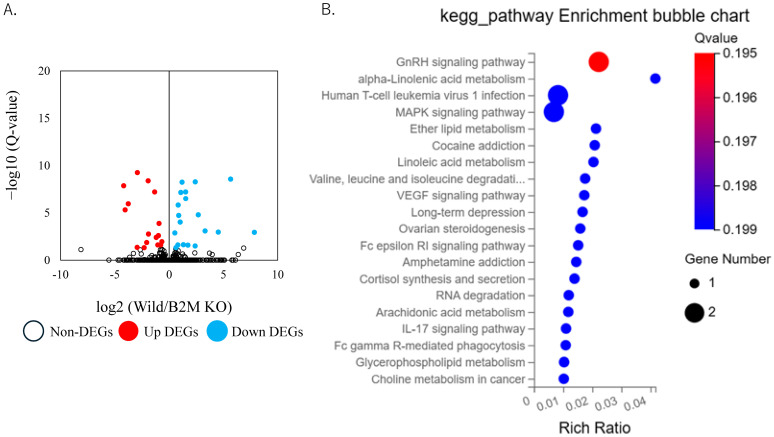
Transcriptomic changes in the sciatic nerve at day 7 post-injury. (**A**) Volcano plot depicting differentially expressed genes (DEGs) in the sciatic nerve at day 7 post-injury. Light blue dots represent genes significantly downregulated in B2M-KO mice compared to C57BL/6J (wild) mice; red dots indicate genes significantly upregulated in B2M-KO mice; and white dots denote non-differentially expressed genes. (**B**) KEGG pathway enrichment analysis of genes significantly downregulated in B2M-KO mice at day 7 post-injury.

At day 7 post-injury, RNA sequencing identified 20 upregulated and 18 downregulated DEGs in B2M-KO mice relative to WT controls (Figure 4A, Supplementary Table S2). Mfap1a remained significantly downregulated. KEGG pathway enrichment analysis at this later time point revealed significant enrichment of pathways associated with tissue remodeling, including “Cardiac muscle contraction”, “Hypertrophic cardiomyopathy,” “Dilated cardiomyopathy”, and “Arginine and proline metabolism”, as well as additional pathways related to immune processes (Figure 4B).

DEGs were identified using a threshold of Q-value < 0.01 and log_2_ fold change ≥ 0.5. KEGG analyses were performed using the BGI online analysis platform (Dr. Tom).

### 3.3. Non-ECM Transcriptomic Changes Following Sciatic Nerve Injury

To assess B2M-dependent regulation beyond the ECM, we extracted the top 10 most downregulated non-ECM transcripts at days 3 and 7 (Table 3). At day 3, the predominant transcripts included *B2m*, *Eif3j1*, *H2-Q7*, *Spint1*, *Jmjd7*, *Pla2g4b*, *AA467197*, *Dlk1*, *Igf2*, and *Ivd*. At day 7, suppression persisted for *B2m*, *Eif3j1*, *Jmjd7*, *Spint1*, and *AA467197*, with additional downregulation of *Gm4841*, *Gbp10*, *Gdf3*, *Pla2g4b*, and *Pttg1*.

### 3.4. Validation of THBS1 Expression by qPCR

**Figure 5:**
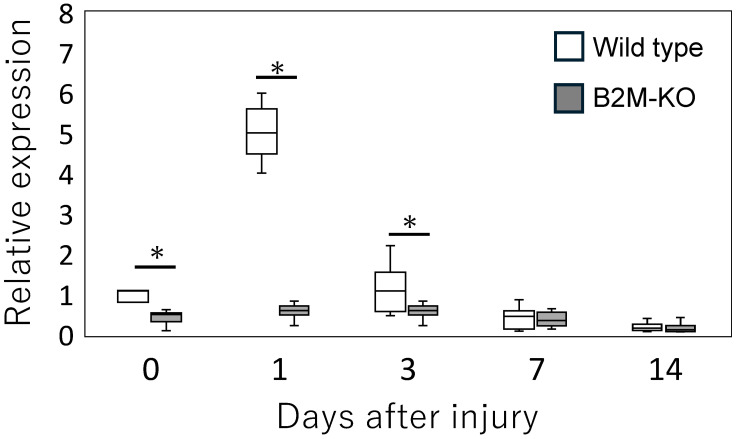
Temporal expression of *Thbs1* in the sciatic nerve following injury. Quantitative real-time PCR analysis of *Thbs*1 mRNA expression in the sciatic nerve of wild-type and B2M-KO mice at days 0, 1, 3, 5, 7, and 14 post-injury. Data are presented as box-and-whisker plots indicating the median, interquartile range, and minimum-to-maximum values. Statistical significance was determined using the Mann–Whitney U test for comparisons between wild-type and B2M-KO mice at each time point. Significant differences between wild-type and B2M-KO mice at the same time point are indicated by asterisks: * *p* < 0.05.

We selected Thbs1 for validation based on its well-established role in extracellular matrix remodeling and its functional involvement in injury-associated processes, including TGF-β activation and modulation of immune responses [10]. Although other ECM-related genes were also downregulated in the RNA-seq dataset, Thbs1 was chosen as a representative target due to its biological relevance and consistent detectability by qPCR. To further investigate the temporal dynamics of Thbs1 expression, we conducted qPCR analyses at multiple time points following sciatic nerve injury. Consistent with the RNA-seq findings, Thbs1 expression was significantly higher in wild-type mice compared with B2M-KO mice at day 1 (*p* < 0.001) and day 3 (*p* = 0.019) post-injury (Figure 5). However, no significant differences were observed between the two groups at day 7 (*p* = 0.772) orday 14 (*p* = 0.436).

## 4. Discussion

Peripheral nerve injury induces a cascade of immune, structural, and molecular responses that shape the tissue environment and influence downstream outcomes. In this study, we investigated the role of β2-microglobulin (B2M), a key component of MHC class I molecules, in regulating extracellular matrix (ECM) gene expression during the early phase following sciatic nerve injury. Transcriptomic analysis revealed that B2M deficiency led to the downregulation of multiple ECM-associated genes, including *Col12a1*, *Col14a1*, *Col6a2*, *Fbn1*, *Ltbp4*, *Mfap1a*, and *Thbs1*, at 3 days post-injury. These genes are functionally involved in fibril organization, Schwann cell adhesion, growth factor sequestration, and axonal guidance. Importantly, these changes occurred prior to the accumulation of CD8^+^ T cells, suggesting a CD8^+^ T-cell-independent regulatory role for B2M during the early stages of peripheral nerve injury.

Peripheral nerve injury is a complex process involving immune activation, ECM remodeling, and neuronal–intrinsic mechanisms. In this study, we demonstrate that B2M, traditionally known for its role in MHC class I assembly and antigen presentation, plays a broader role in modulating the regenerative microenvironment, particularly through regulation of ECM-related genes and signaling pathways. Transcriptomic profiling revealed a coordinated downregulation of key ECM constituents in B2M-KO mice at early post-injury stages. *Col12a1* and *Col14a1* are FACIT collagens (Fibril-Associated Collagens with Interrupted Triple helices) that organize collagen I and III fibrils [23,24], promote Schwann cell migration and effective axonal guidance, and modulate fibril diameter and matrix porosity to fine-tune extracellular signaling gradients for regenerating axons [25]. The major basement membrane collagen Col6a2 supports Schwann cell adhesion and myelination; its deficiency has been linked to delayed remyelination in nerve injury models [8]. *Fbn1*, encoding fibrillin-1, assembles elastic microfibrils that provide mechanical resilience and sequester growth factors during regeneration [26]. Ltbp4 binds latent TGF-β complexes to the ECM, interacting with fibrillin and collagen assemblies; fibrillin-1 deficiency reduces LTBP-4 accumulation [23]. Mfap1a belongs to the microfibril-associated protein (MFAP) family of ECM glycoproteins, playing roles in microfibril assembly, elastinogenesis, and tissue homeostasis. [27]. Thbs1 (thrombospondin-1) is a matricellular protein that interacts with integrins and CD47 to promote neurite outgrowth and synaptogenesis; Thbs1 overexpression enhances retinal ganglion cell regeneration and likely exerts similar effects in peripheral nerves [10]. Thbs3, though less characterized, shares structural domains with Thbs1, and its expression is elevated under inflammatory conditions in the central nervous system [28]. Our results suggest a direct role of B2M in establishing a pro-regenerative ECM landscape.

The relevance of our findings is supported by previous studies conducted in other neural systems. Tang et al. demonstrated that the downregulation of Thbs1 impairs nerve cell survival and disrupts both PI3K-Akt and TGF-β signaling pathways in entrectinib-treated models [29]. Conversely, Thbs1 overexpression was shown to restore these pathways and promote neuronal survival. Similarly, Bray et al. identified Thbs1 as a key regulator of axon regeneration in retinal ganglion cells [10]. These findings align with our results and support the hypothesis that B2M-dependent *Thbs1* expression may play a pivotal role in nerve repair. The modulation of PI3K-Akt and TGF-β signaling through Thbs1 may represent a conserved mechanism contributing to axonal regeneration across distinct neural contexts. While CD8^+^ T cells have been implicated in both protective and detrimental roles following nerve injury, the precise mechanisms of their recruitment and activation remain incompletely understood. Zhou et al. highlighted an aging-dependent decline in neuronal regeneration driven by CD8^+^ T-cell–neuron crosstalk and caspase-3 activation [30]. Similarly, studies by Hu et al. and Davies et al. reported CD8^+^ T-cell infiltration [31,32]. However, given that MHC class I expression remains low in injured sensory neurons [33], the trigger for CD8^+^ T-cell activation in this context is unclear. Our observation that ECM-related gene suppression occurs before CD8^+^ T-cell accumulation suggests that B2M may have early, CD8^+^ T-cell-independent effects critical for setting the stage for subsequent immune–neuronal interactions.

In addition to ECM alterations, B2M deficiency led to the suppression of *Spint1* and *Igf2*, highlighting protease regulation and growth factor signaling pathways critical for nerve repair. Spint1, a serine protease inhibitor controlling HGF activation [34], may impair HGF-driven Schwann cell proliferation and axonal elongation when downregulated, as HGF promotes peripheral nerve regeneration [35]. IGF2 has been extensively documented to enhance peripheral nerve repair: sustained delivery of IGF2 in a rat sciatic nerve crush model significantly increased axonal regeneration distance, whereas anti-IGF2 serum treatment inhibited regeneration [36]. In cultured neuron models, IGF2 supplementation promoted neurite outgrowth and survival in sympathetic and sensory neuron [37]. The observed downregulation of *Igf2* in B2M-KO mice may therefore contribute to delayed axonal elongation and reduced neuronal viability, identifying IGF2 as a key mediator of B2M-dependent regenerative support. Taken together, our study broadens the functional landscape of B2M by demonstrating its involvement in ECM remodeling and regenerative signaling pathways following peripheral nerve injury. These findings position B2M not only as a mediator of immune cell function but also as a key orchestrator of the molecular milieu that supports tissue regeneration.

While our study provides comprehensive transcriptomic insights, several limitations merit discussion. First, bulk RNA-seq cannot distinguish cell type-specific transcriptional changes; single-cell RNA-seq or spatial transcriptomics would refine our understanding of Schwann cell, fibroblast, macrophage, and endothelial contributions. Second, protein-level validation is necessary to confirm that mRNA alterations translate to functional protein changes; future work will employ mass spectrometry-based proteomics and immunohistochemical analyses. We did not assess protein expression of key ECM or non-ECM targets in this study. Third, we did not perform histological examinations (e.g., myelin staining or immunofluorescence of ECM proteins) to evaluate structural remodeling of the injured nerve. Fourth, direct measures of regeneration—such as nerve conduction velocity, axon morphometry, and behavioral/functional recovery assays—were beyond the scope of this work but are critical for linking molecular findings to physiological outcomes. Fifth, we employed a CCI model of the sciatic nerve, which induces persistent partial nerve damage and is widely used to study neuropathic pain and injury-associated molecular responses. However, this model does not permit full axonal regeneration due to ongoing compression, and the molecular alterations observed in this study likely reflect injury responses and partial remodeling rather than true regeneration. To fully elucidate the role of B2M in regenerative processes, future investigations using models that support complete nerve regeneration—such as nerve crush or transection with end-to-end repair—will be essential. Lastly, our use of global B2M knockout mice precludes delineation of cell-intrinsic versus systemic effects; conditional, cell-specific B2M ablation will be important to clarify mechanistic pathways.

## 5. Conclusions

In this study, we investigated the role of B2M in peripheral nerve injury using a CCI model in wild-type and B2M-KO mice. Through transcriptomic and flow cytometric analyses, we found that B2M deficiency led to early downregulation of key ECM-related genes—including *Col12a1*, *Col14a1*, *Col6a2*, *Fbn1*, *Ltbp4*, *Mfap1a*, and *Thbs1*—prior to the accumulation of CD8^+^ T cells. These findings suggest that B2M plays a CD8^+^ T-cell-independent role in shaping the ECM landscape during peripheral nerve injury. Our results position B2M as a molecular link between immune modulation and structural remodeling in the injured peripheral nervous system. From a clinical perspective, targeting B2M-regulated ECM pathways may offer a novel strategy to enhance tissue remodeling and functional recovery following nerve damage. Future studies incorporating protein-level validation, functional assays, and regenerative models will be essential to evaluate the therapeutic potential of modulating B2M-dependent signaling in peripheral neuropathies.

## Figures and Tables

**Table 1:** Sequences of the primers used in this study.

Primer	Sequence (5′–3′)	Product Size (bp)
Thbs1-F	TAGCTGGAAATGTGGTGCGT	123
Thbs1-R	TTGCACCGATGTTCTCCGTT	
Gapdh-F	AACTTTGGCATTGTGGAAGG	223
Gapdh-R	ACACATTGGGGGTAGGAACA	

**Table 2:** ECM-related genes downregulated in B2M-KO mice at day 3 post-injury.

Gene ID	Gene Symbol	log_2_ (FC)	Q-Value
12816	*Col12a1*	−0.584	6.27 × 10^−3^
12818	*Col14a1*	−0.593	3.37 × 10^−4^
14118	*Fbn1*	−0.530	3.18 × 10^−3^
108075	*Ltbp4*	−0.500	4.58 × 10^−2^
67532	*Mfap1a*	−1.094	6.25 × 10^−6^
17390	*Mmp2*	−0.610	2.89 × 10^−3^
21825	*Thbs1*	−0.568	2.89 × 10^−3^
21827	*Thbs3*	−0.581	2.60 × 10^−3^
21923	*Tnc*	−0.583	1.93 × 10^−4^
81877	*Tnxb*	−0.795	3.12 × 10^−6^

**Table 3:** Top 10 downregulated non-ECM DEGs following sciatic nerve injury.

Day 3				Day 7			
Gene ID	Gene Symbol	log2 (FC)	Q-Value	Gene ID	Gene Symbol	log2 (FC)	Q-Value
12010	*B2m*	−6.752	1.5 × 10^−301^	12010	*B2m*	−7.459	8.4 × 10^−140^
78655	*Eif3j1*	−6.601	5.9 × 10^−82^	78655	*Eif3j1*	−6.853	1.2 × 10^−60^
15018	*H2-Q7*	−6.407	7.5 × 10^−10^	433466	*Jmjd7*	−4.193	1.4 × 10^−8^
20732	*Spint1*	−4.317	8.9 × 10^−10^	225594	*Gm4841*	−4.057	4.8 × 10^−6^
433466	*Jmjd7*	−3.954	1.9 × 10^−7^	20732	*Spint1*	−3.762	1.1 × 10^−6^
211429	*Pla2g4b*	−2.181	3.2 × 10^−2^	626578	*Gbp10*	−2.944	4.4 × 10^−2^
433470	*AA467197*	−1.734	2.0 × 10^−4^	433470	*AA467197*	−2.922	5.6 × 10^−10^
13386	*Dlk1*	−1.480	3.5 × 10^−3^	14562	*Gdf3*	−2.298	4.7 × 10^−2^
16002	*Igf’2*	−1.411	4.0 × 10^−3^	211429	*Pla2g4b*	−2.070	1.4 × 10^−2^
56357	*Ivd*	−1.062	8.8 × 10^−6^	30939	*Pttg1*	−1.935	4.1 × 10^−9^

## Data Availability

The original contributions presented in this study are included in the article. Further inquiries can be directed to the corresponding author.

## Supplementary Materials

The following supporting information can be downloaded at: https://www.mdpi.com/article/10.3390/neurosci6030059/s1, Table S1: DEGs in B2M KO mice compared to wild-type mice at 3 days after sciatic nerve injury; Table S2: DEGs in B2M KO mice compared to wild-type mice at 7 days after sciatic nerve injury.

## Abbreviations

The following abbreviations are used in this manuscript:

ECM	extracellular matrix
B2M	β2-microglobulin
TGF-β	transforming growth factor-beta
CCI	chronic constriction injury

## References

[1] Robinson L.R. (2022). Traumatic injury to peripheral nerves. Muscle Nerve.

[2] Taylor C.A., Braza D., Rice J.B., Dillingham T. (2008). The incidence of peripheral nerve injury in extremity trauma. Am. J. Phys. Med. Rehabil..

[3] Grosu-Bularda A., Vancea C.V., Hodea F.V., Cretu A., Bordeanu-Diaconescu E.M., Dumitru C.S., Ratoiu V.A., Teodoreanu R.N., Lascar I., Hariga C.S. (2025). Optimizing Peripheral Nerve Regeneration: Surgical Techniques, Biomolecular and Regenerative Strategies-A Narrative Review. Int. J. Mol. Sci..

[4] Du J., Cheng N., Deng Y., Xiang P., Liang J., Zhang Z., Hei Z., Li X. (2023). Astrocyte senescence-like response related to peripheral nerve injury-induced neuropathic pain. Cell Mol. Biol. Lett..

[5] Liu L., Chen J., Yin W., Gao P., Fan Y., Wen D., Jiao Y., Yu W. (2024). The peripheral Atf3 (+) neuronal population is responsible for nerve regeneration at the early stage of nerve injury revealed by single-cell RNA sequencing. Acta Biochim. Biophys. Sin..

[6] Yong N., Guoping C. (2009). The role and mechanism of the up-regulation of fibrinolytic activity in painful peripheral nerve injury. Neurochem. Res..

[7] Wei G., Chen C., Li X., Wang H., Li Z., Gou X., Zhang P. (2025). In situ piezoelectricity induces M2 polarization of macrophages to regulate Schwann cells for alleviating neuropathic pain of CCI rats. Biomater. Adv..

[8] Chen P., Cescon M., Megighian A., Bonaldo P. (2014). Collagen VI regulates peripheral nerve myelination and function. FASEB J..

[9] Chen P., Cescon M., Bonaldo P. (2015). The Role of Collagens in Peripheral Nerve Myelination and Function. Mol. Neurobiol..

[10] Bray E.R., Yungher B.J., Levay K., Ribeiro M., Dvoryanchikov G., Ayupe A.C., Thakor K., Marks V., Randolph M., Danzi M.C. (2019). Thrombospondin-1 Mediates Axon Regeneration in Retinal Ganglion Cells. Neuron.

[11] Sang Q., Sun D., Chen Z., Zhao W. (2018). NGF and PI3K/Akt signaling participate in the ventral motor neuronal protection of curcumin in sciatic nerve injury rat models. Biomed. Pharmacother..

[12] Jalise S.Z., Habibi S., Fath-Bayati L., Habibi M.A., Ababzadeh S., Hosseinzadeh F. (2024). Role and Interplay of Different Signaling Pathways Involved in Sciatic Nerve Regeneration. J. Mol. Neurosci..

[13] Ding Z., Jiang M., Qian J., Gu D., Bai H., Cai M., Yao D. (2024). Role of transforming growth factor-beta in peripheral nerve regeneration. Neural Regen. Res..

[14] Koller B.H., Marrack P., Kappler J.W., Smithies O. (1990). Normal development of mice deficient in beta 2M, MHC class I proteins, and CD8+ T cells. Science.

[15] Neefjes J., Jongsma M.L., Paul P., Bakke O. (2011). Towards a systems understanding of MHC class I and MHC class II antigen presentation. Nat. Rev. Immunol..

[16] Oliveira A.L., Thams S., Lidman O., Piehl F., Hokfelt T., Karre K., Linda H., Cullheim S. (2004). A role for MHC class I molecules in synaptic plasticity and regeneration of neurons after axotomy. Proc. Natl. Acad. Sci. USA.

[17] Muneshige K., Onuma K., Sukegawa K., Otake Y., Inoue G., Takaso M., Uchida K. (2022). beta2-Microglobulin Elevates COL5A1 mRNA in the Subsynovial Connective Tissue of Patients Receiving Hemodialysis with Carpal Tunnel Syndrome. Cureus.

[18] Li D., Zhang Q., Li L., Chen K., Yang J., Dixit D., Gimple R.C., Ci S., Lu C., Hu L. (2022). beta2-Microglobulin Maintains Glioblastoma Stem Cells and Induces M2-like Polarization of Tumor-Associated Macrophages. Cancer Res..

[19] Sun W., Gui L., Zuo X., Zhang L., Zhou D., Duan X., Ren W., Xu G. (2016). Human epithelial-type ovarian tumour marker beta-2-microglobulin is regulated by the TGF-beta signaling pathway. J. Transl. Med..

[20] Ma C.H., Omura T., Cobos E.J., Latremoliere A., Ghasemlou N., Brenner G.J., van Veen E., Barrett L., Sawada T., Gao F. (2011). Accelerating axonal growth promotes motor recovery after peripheral nerve injury in mice. J. Clin. Investig..

[21] Nishimoto S., Okada K., Tanaka H., Okamoto M., Fujisawa H., Okada T., Naiki M., Murase T., Yoshikawa H. (2016). Neurotropin attenuates local inflammatory response and inhibits demyelination induced by chronic constriction injury of the mouse sciatic nerve. Biologicals.

[22] Kleinschnitz C., Hofstetter H.H., Meuth S.G., Braeuninger S., Sommer C., Stoll G. (2006). T cell infiltration after chronic constriction injury of mouse sciatic nerve is associated with interleukin-17 expression. Exp. Neurol..

[23] Ricard-Blum S., Ruggiero F. (2005). The collagen superfamily: From the extracellular matrix to the cell membrane. Pathol. Biol..

[24] Chiquet M., Birk D.E., Bonnemann C.G., Koch M. (2014). Collagen XII: Protecting bone and muscle integrity by organizing collagen fibrils. Int. J. Biochem. Cell Biol..

[25] Sances S., Ho R., Vatine G., West D., Laperle A., Meyer A., Godoy M., Kay P.S., Mandefro B., Hatata S. (2018). Human iPSC-Derived Endothelial Cells and Microengineered Organ-Chip Enhance Neuronal Development. Stem Cell Rep..

[26] Chernousov M.A., Baylor K., Stahl R.C., Stecker M.M., Sakai L.Y., Lee-Arteaga S., Ramirez F., Carey D.J. (2010). Fibrillin-2 is dispensable for peripheral nerve development, myelination and regeneration. Matrix Biol..

[27] Zhu S., Ye L., Bennett S., Xu H., He D., Xu J. (2021). Molecular structure and function of microfibrillar-associated proteins in skeletal and metabolic disorders and cancers. J. Cell Physiol..

[28] Ching S., Zhang H., Chen Q., Quan N. (2007). Differential expression of extracellular matrix and adhesion molecule genes in the brain of juvenile versus adult mice in responses to intracerebroventricular administration of IL-1. Neuroimmunomodulation.

[29] Tang Q., Dong J., Zhang F., Zhao D., Yang Q., Wen J., Sun Y., Wei J., Liu Z. (2025). Entrectinib can induce nerve cell damage by inhibiting PI3K-AKT and TGF-beta signaling pathways. Front. Pharmacol..

[30] Zhou L., Kong G., Palmisano I., Cencioni M.T., Danzi M., De Virgiliis F., Chadwick J.S., Crawford G., Yu Z., De Winter F. (2022). Reversible CD8 T cell-neuron cross-talk causes aging-dependent neuronal regenerative decline. Science.

[31] Bali K.K., Kuner R. (2017). Therapeutic potential for leukocyte elastase in chronic pain states harboring a neuropathic component. Pain.

[32] Hu P., Bembrick A.L., Keay K.A., McLachlan E.M. (2007). Immune cell involvement in dorsal root ganglia and spinal cord after chronic constriction or transection of the rat sciatic nerve. Brain Behav. Immun..

[33] Bombeiro A.L., Thome R., Oliveira Nunes S.L., Monteiro Moreira B., Verinaud L., Oliveira A.L. (2016). MHC-I and PirB Upregulation in the Central and Peripheral Nervous System following Sciatic Nerve Injury. PLoS ONE.

[34] Kawaguchi T., Qin L., Shimomura T., Kondo J., Matsumoto K., Denda K., Kitamura N. (1997). Purification and cloning of hepatocyte growth factor activator inhibitor type 2, a Kunitz-type serine protease inhibitor. J. Biol. Chem..

[35] Ko K.R., Lee J., Lee D., Nho B., Kim S. (2018). Hepatocyte Growth Factor (HGF) Promotes Peripheral Nerve Regeneration by Activating Repair Schwann Cells. Sci. Rep..

[36] Near S.L., Whalen L.R., Miller J.A., Ishii D.N. (1992). Insulin-like growth factor II stimulates motor nerve regeneration. Proc. Natl. Acad. Sci. USA.

[37] Recio-Pinto E., Rechler M.M., Ishii D.N. (1986). Effects of insulin, insulin-like growth factor-II, and nerve growth factor on neurite formation and survival in cultured sympathetic and sensory neurons. J. Neurosci..

